# Comparative Evaluation of Dentinal Crack After Root Canal Preparation Using TruNatomy, Neoendo Flex, and Neoendo Neohybrid Files: An In Vitro Study

**DOI:** 10.7759/cureus.49593

**Published:** 2023-11-28

**Authors:** Priyanka Priyadarshni, Ajay Nagpal, Arina Arif, Abhishek Sharma, Mutiur Rahman, Shreyasi Sinha

**Affiliations:** 1 Department of Conservative Dentistry and Endodontics, Kanti Devi Dental College and Hospital, Mathura, IND

**Keywords:** root canal preparation, neoendo neohybrid, neoendo flex, trunatomy, dentinal cracks

## Abstract

Aim: The objective of the study was to assess and compare the dentinal microcracks produced by TruNatomy, Neoendo Flex, and Neoendo Neohybrid files during root canal preparation.

Material and methods: In this in vitro investigation, four groups of 25 samples each were assembled from 100 mandibular premolar teeth. Using TruNatomy, Neoendo Flex, and Neoendo Neohybrid files, or leaving the teeth unprepared (control), the teeth underwent root canal preparation. Horizontal sections were taken at different distances from the apex to analyze dentinal cracks. A stereomicroscope was used to assess the existence or lack of cracks, and chi-square tests were performed on the data.

Results: In the control group, there were no cracks. TruNatomy files created fewer cracks compared to Neoendo Flex and Neoendo Neohybrid files.

Conclusions: This in vitro investigation demonstrates that the root surface might acquire dentinal cracks as a result of nickel-titanium instruments. Compared to Neoendo Flex and Neoendo Neohybrid files, TruNatomy showed a decreased incidence of cracks. However, it is important to note that these conclusions are limited to the scope of this study.

## Introduction

Dentinal injury to roots during endodontic therapy is quite possible. Different factors, such as the physical characteristics of the teeth, the method of tooth preparation, and instrumentation using rotating nickel-titanium (NiTi) instruments, may have an impact on the quantity of dentinal damage [1].

Well-known problems include things like transportation, ledge creation, and perforation. The root dentin, however, could potentially fracture or develop craze lines as a result of the preparation techniques. Root dentin may show signs of vertical root fracture and crack development both during and after endodontic therapy [2].

One of the irritating side effects of root canal therapy, which frequently necessitates tooth extraction, is a vertical root fracture. Because of their extremely elastic behavior, NiTi rotary files have gained popularity as they are the best option for contouring asymmetric root canals. That is mostly due to the extremely elastic NiTi alloy, which gives the instruments more flexibility and efficiently follows the actual course of the root canal. The clinician must therefore be aware of the advantages and disadvantages of each endodontic file system in use today [3,4].

TruNatomy (TRN) files by Dentsply Sirona (Charlotte, NC, United States) are advanced endodontic tools known for their heat-treated NiTi wire, which provides enhanced flexibility and pre-curvature capability. They have a slim wire design and an off-centered cross-section. TruNatomy excels in debridement, debris removal, and preservation of natural canal anatomy. Its smaller flute diameter of 0.8 mm reduces the risk of file separation compared to generic variable taper files. TruNatomy maintains structural integrity and offers increased resistance to cyclic fatigue [5].

Neoendo Flex files by Orikam (Gurgaon, Haryana, India) are innovative rotary endodontic files with enhanced flexibility and efficient cutting. They exhibit excellent fatigue resistance, making them highly effective for endodontic procedures [6].

Orikam's Neoendo Neohybrid files are a recent addition, offering asymmetric movement and two-point contact with the canal wall. They provide enhanced flexibility for shaping curved canals and efficient debris removal while maintaining cutting efficiency [7].

Consequently, this research aimed to analyze and contrast the prevalence of dentinal microcracks produced by the use of rotary NiTi files, especially TruNatomy, Neoendo Flex, and Neoendo Neohybrid, during root canal procedures.

## Materials and methods

Materials

The materials used were as follows: (1) 100 freshly extracted human mandibular premolar teeth; (2) Digital Vernier caliper (Sudershan Measuring and Engineering Pvt. Ltd., New Delhi, India); (3) Radiovisiography (RVG) (Satelec and Pierre Ronald Bordeaux, Merignac, France); (4) Thymol Crystals (VDH Organics Pvt. Ltd., Ghaziabad, India); (5) Physiologic saline (Swaroop Pharmaceuticals Ltd., Aligarh, India); (6) Conventional hand K-files 10/.02, 15/.02, 20/.02 (DENTSPLY, Maillefer, Ballaigues, Switzerland); (7) Rotary files: (a) TruNatomy (DENTSPLY, Maillefer, Ballaigues, Switzerland), (b) Neoendo Flex (Orikam Healthcare, Haryana, India), and (c) Neoendo Neohybrid (Orikam Healthcare, Haryana, India); (8) Dental operating microscope (DOM) (Roslane Meditech, Haryana, India); (9) Acrylic resin (Nimai Polymars, Roorkee, India); (10) Aluminum foil (ME Foil, Parekh Aluminex Ltd., Dadra, India); (11) Light body hydrophilic vinyl polysiloxane impression material (Coltene PRESIDENT light body, Switzerland); (12) Ethylenediaminetetraacetic acid (EDTA) (Smart Prep, safe endo); (13) 2.5%NaOCL (Chemident); (14) 27 Gauge needle and syringe (5 mL) (Dispovan); (15) Isomet slow speed precision sectioning saw (Buehler Ltd., Waukegan Road, Lake Bluff, IL, United States); (16) Stereomicroscope (Expert DN; Muller Optronic, Erfurt, Germany); and (17) X-smart plus rotary system (DENTSPLY, Maillefer, Ballaigues, Switzerland).

Sample preparation

The Institutional Ethical Committee of Kanti Devi Dental College and Hospital, Mathura, Uttar Pradesh, has issued approval (reference number KDDC/Admn/16237/2023) for this study. After obtaining ethical approval, 100 freshly extracted human mandibular premolars, extracted for periodontal or orthodontic reasons, were selected for this study. All teeth were cleaned of tissue fragments and visible debris using an ultrasonic scaler (EMS, Switzerland) and stored in a thymol solution at room temperature until use. All teeth were radiographed in buccolingual and mesiodistal directions to confirm the existence of fully formed apex, single apical foramen, no signs of internal resorption, no pulp stones, root canal calcification, obstruction, or previous endodontic therapy. The roots with a curvature of <5˚ and a completely formed apex with patent foramina were selected. The external root surfaces were examined under a DOM at 20× magnification to exclude any visible root caries, external resorption, fractures, or cracks. Teeth that deviated from such findings were excluded from the study and replaced with new teeth.

A standardized root length of 12±1 mm was achieved by separating the tooth crowns at the cementoenamel junction using an Isomet saw and water coolant. To make sure there were no defects, root samples were carefully inspected under a dental operating microscope. An ISO No. 10 K-file was used to verify canal patency after the creation of access cavities. By visualizing a size No. 10 K-file placed at the apical foramen and reducing 1 mm, the dimension that could be achieved was decided.

Aluminum foil-wrapped roots were inserted in acrylic resin in a petroleum jelly-coated syringe barrel to represent the periodontal ligament. After the roots had been taken out, the foil was peeled off, and the periodontal ligament gaps were reproduced employing a hydrophilic polyvinyl siloxane impression material. After that, the roots were inserted into the acrylic resin. Twenty-five of the 100 samples were selected as the control group and were not prepared. Using the instrumentation techniques, the remaining 75 samples were categorized into three groups of 25 each: Group I-TruNatomy, Group II-Neoendo Flex, and Group III-Neoendo Neohybrid. In all three groups, EDTA gel was applied as a lubricant during the canal instrumentation procedures.

Preparation of canal

At first, size No. 10 K-files were utilized to negotiate the canals. The working length had been established by advancing the file until it was barely noticeable at the apical foramen and deducting 1 mm after any gross pulpal tissue was removed. Following that, rotary instruments were employed with a torque-limited endo motor (X-Smart Plus, Dentsply Tulsa Dental) attached to a handpiece with a 16:1 reduction. For each file, the recommended rotational speed and torque limit were maintained. The stated protocol was followed in the preparation of the canals, which included irrigation with 2 mL of 3% sodium hypochlorite solution. After preparation, 5 mL of distilled water were used to rinse the samples from the prepared groups.

Group I was prepared with TruNatomy in the sequence of TruNatomy Orifice Modifier, TruNatomy Glide, TruNatomy Small, and TruNatomy Prime at a speed of 500 rpm and torque of 1.5N Cm up to size 26-0.4% taper. Group II was prepared with Neoendo Flex at a speed of 350 rpm and torque of 1.5 NCm up to size 25-0.4% taper. Group III was prepared with Neoendo Neohybrid at a speed of 350 rpm and torque of 1.5 NCm up to size 25-0.4% taper. In Group IV (Control Group), root canal shaping was not performed.

Sectioning and microscopic examination

All the roots were sectioned horizontally at 3, 6, and 9 mm from the apex using a low-speed Isomet saw under water cooling to examine micro-dentin cracks in the apical third, middle third, and coronal third of the root, a total of 75 sections in each group. Digital images of each section were captured at 25× magnifications using a digital stereomicroscope. Microscopic examinations of specimens were done to examine micro-dentin cracks. In order to avoid confusing definitions of root fractures, root dentin defects were classified as no defect, craze line, partial crack, or a complete crack [8].

Types of root dentinal cracks

No Defect

**Figure 1:**
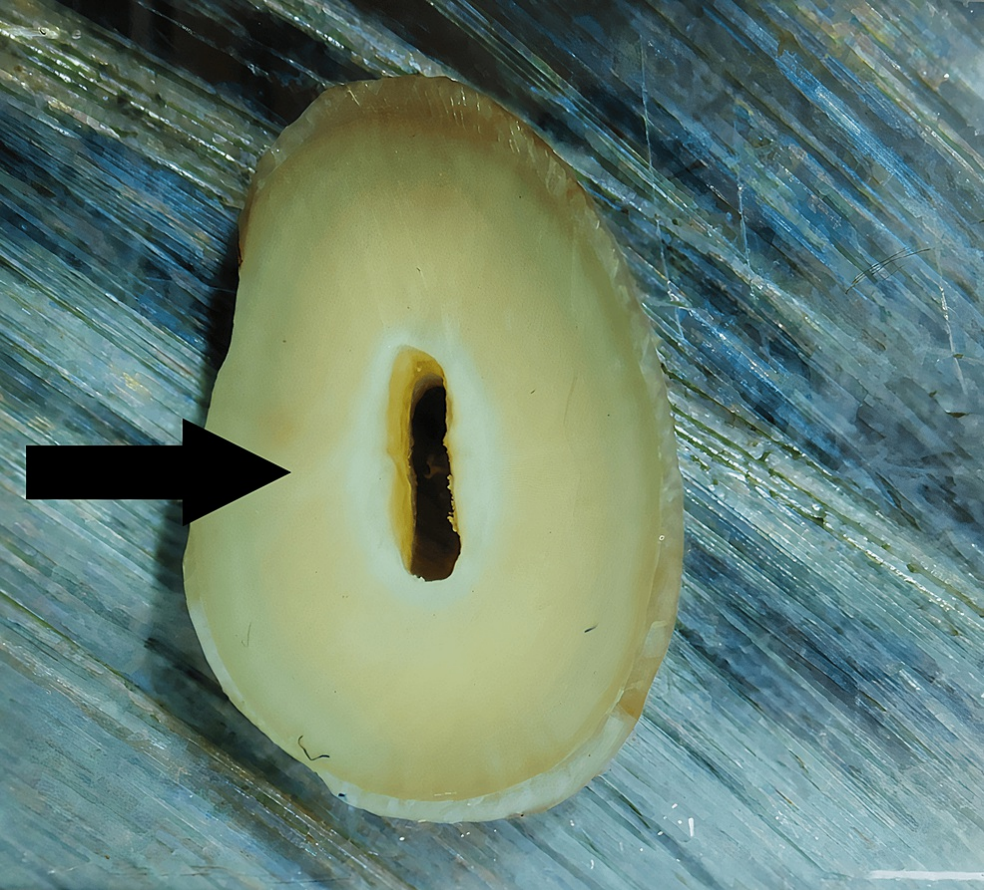
No defect: No lines or cracks within the root dentin. The arrow shows the absence of defect on the root surface.

Neither internally within the root canal wall nor externally on the surface of the root, the dentin showed no signs of cracks or craze lines (Figure 1).

Craze Line

**Figure 2:**
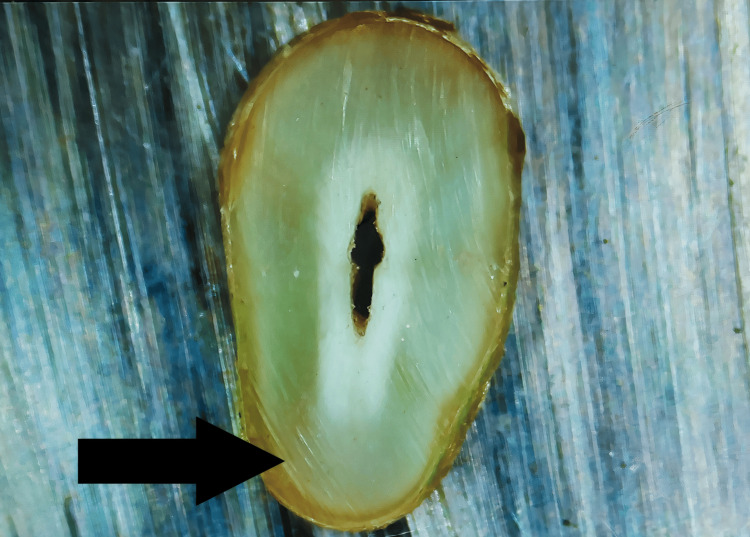
Craze line: Line that reaches the dentin but does not extend into the canal lumen. The arrow shows the presence of craze line on the root surface.

Although it does not cross the canal lumen, a line can be observed extending from the outer canal walls into the dentin (Figure 2).

Partial Crack

**Figure 3:**
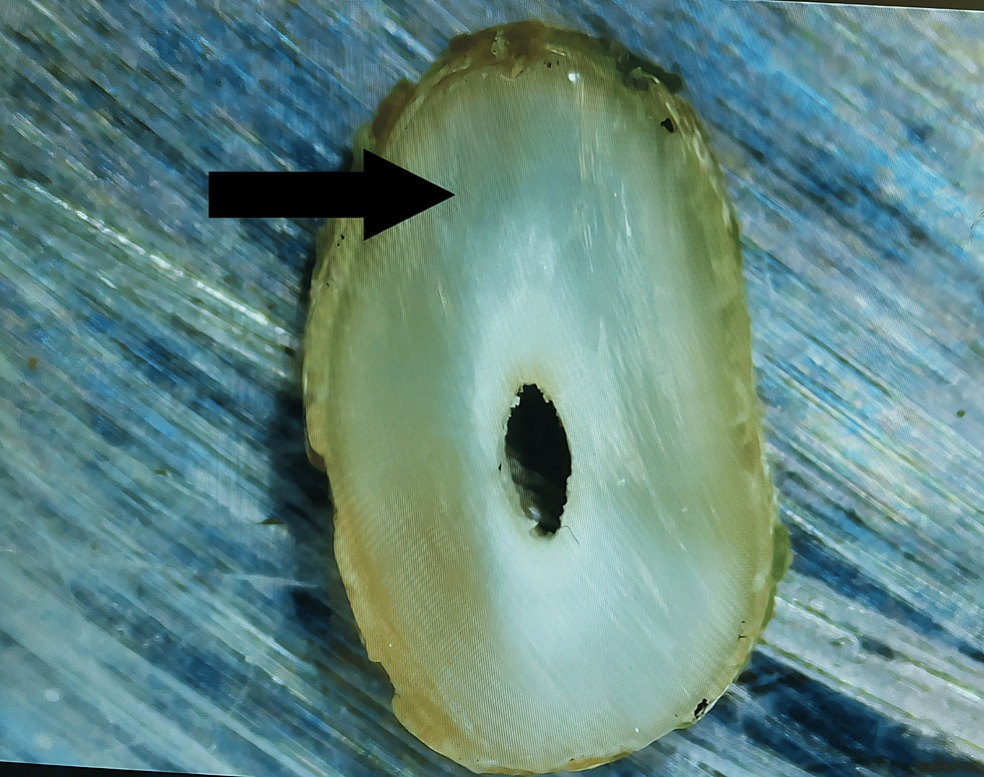
Partial crack: Line that pierces the dentin but does not reach the outer surface of the canal walls. The arrow shows the presence of partial crack on the root surface.

The dentin is visible along a line that runs from the canal walls, but it does not continue to the exterior surface of the root (Figure 3).

Complete Crack

**Figure 4:**
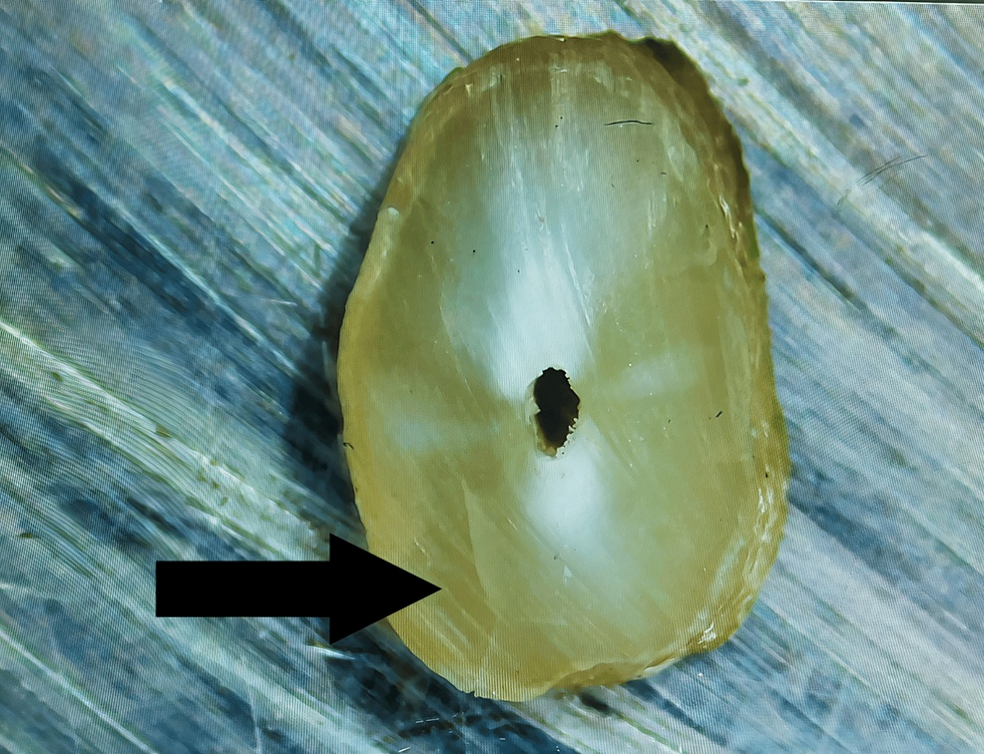
Fracture: Line leading from the outer surface of the root portions to the root canal space. The arrow shows the presence of fracture on the root surface.

From the inner root canal space to the exterior surface of the root, a straight line can be seen (Figure 4). 

Statistical analysis

The findings were analyzed using the chi-square test with a significance level of P<0.05. Statistical analysis was conducted using IBM SPSS Statistics for Windows, Version 22.0 for Windows (Released 2013; IBM Corp., Armonk, New York, United States).

## Results

The different filing methods were compared based on the presence and absence of defects in each third of the sample. In the coronal third, there were no dentinal defects found in all 25 samples of Group I (TruNatomy) and Group IV (control). Defects were seen in two samples (8%) in Group II (Neoendo Flex), while the maximum number of root dentinal defects (six samples (24%)) was found in Group III (Neoendo Neohybrid). The chi-square test showed a highly significant difference between the groups, with a chi-square value of 13.04 and a P-value of 0.005.

The pairwise comparison of groups in coronal third showed a significant difference between Group 1 versus Group 3 and Group 3 versus Group 4. There were non-significant difference between Group 1, Group 2, and Group 4. Therefore, the root dentinal defect in the coronal third was Group 1 = Group 4 ≈ Group 2 < Group 3 (Table 1).

**Table 1:** Root dentinal defects at the coronal third were pairwise compared between different groups. Group I: TruNatomy; Group II: Neoendo Flex; Group III: Neoendo Neohybrid; Group IV: control group. *Significant.

Pair of groups	Chi-square value	P-value
Group I versus Group II	2.08	0.490
Group I versus Group III	6.81	0.022*
Group I versus Group IV	0.04	0.011
Group II versus Group III	2.38	0.247
Group II versus Group IV	2.08	0.490
Group III versus Group IV	6.81	0.022*

The middle third of samples showed only a root dentinal defect in one sample (4%) of Group I (TruNatomy). Defects were seen in six samples (24%) in Group II (Neoendo Flex), while the maximum number of root dentinal defects, i.e., 10 samples (40%), was found in Group III (Neoendo Neohybrid). There were root dentinal defects in two samples (8%) in Group IV (control). The chi-square test showed a highly significant difference between the groups, with a chi-square value of 16.38 and a P-value of 0.001.

The pairwise comparison of groups in the middle third showed a significant difference between Group 1 versus Group 2, Group 1 versus Group 3, Group 2 versus Group 4, and Group 3 versus Group 4. There was a non-significant difference between the other pair of groups. Therefore, the root dentinal defect in the middle third was Group 1 ≈ Group 4 < Group 2 ≈ Group 3 (Table 2).

**Table 2:** Root dentinal defects in the middle third were compared pairwise between the groups. Group I: TruNatomy; Group II: Neoendo Flex; Group III: Neoendo Neohybrid; Group IV: control group. *Significant.

Pair of groups	Chi-square value	P-value
Group I versus Group II	4.15	0.042*
Group I versus Group III	9.44	0.005*
Group I versus Group IV	0.35	1.00
Group II versus Group III	1.47	0.364
Group II versus Group IV	3.78	0.047*
Group III versus Group IV	7.02	0.018*

The apical third of samples showed a root dentinal defect in three samples (12%) of Group I (TruNatomy). Defects were seen in nine samples (36%) in Group II (Neoendo Flex), while the maximum number of root dentinal defects, i.e., 13 samples (52%), were found in Group III (Neoendo Neohybrid). There were root dentinal defects in two samples (8%) in Group IV (Control). The chi-square test showed a highly significant difference between the groups, with a chi-square value of 13.19 and a P-value of 0.004.

The pairwise comparison of groups in the apical third showed a significant difference between Group 1 versus Group 2, Group 1 versus Group 3, Group 2 versus Group 4, and Group 3 versus Group 4. There was a non-significant difference between the other pair of groups. Therefore, the root dentinal defect in the apical third was Group 1 ≈ Group 4 < Group 2 ≈ Group 3 (Table 3).

**Table 3:** Root dentinal defects at the apical third were pairwise compared among groups. Group I: TruNatomy; Group II: Neoendo Flex; Group III: Neoendo Neohybrid; Group IV: control group. *Significant. **Highly significant.

Pair of groups	Chi-square value	P-value
Group I versus Group II	3.94	0.047*
Group I versus Group III	9.19	0.005**
Group I versus Group IV	0.22	1.00
Group II versus Group III	1.29	0.393
Group II versus Group IV	5.71	0.037*
Group III versus Group IV	11.52	0.001**

## Discussion

The "endodontic triangle" (i.e., shaping canals, cleaning in three dimensions, and filling root canal systems) must be strictly followed for any endodontic procedure to be successful. One of the crucial steps in the root canal procedure is the cleaning and shaping of the root canal system. Therefore, reliably formed root canals are necessary for successful endodontic therapy in order to enable three-dimensional obturation [2]. Occasionally, we unavoidably cause damage to the root dentin, which opens the door for infections, such as perforation, zipping, dentinal cracks, and micro delicate fractures, leading to treatment failure [9].

To ensure that the tooth is adequately strong for long-term function, it is also crucial to prevent iatrogenic damage to the root dentin [10].

Compared to hand instruments, NiTi rotary instruments produce even, rounded root canals and more disciplined tapers. They were therefore anticipated to result in a consistent distribution of stress and ultimately less load on the canal walls. However, it was discovered that, in contrast to those prepared with hand instruments, root canals performed with NiTi rotary instruments had a greater prevalence of dentinal defects. This was because rotary instruments had a greater taper and more rotations than hand instruments did [11,12].

Wilcox et al. suggested in a previous study that when more dentine was removed, the roots were more likely to fracture [8]. Stress and strain from using various rotational NiTi endodontic instruments during root canal preparation may cause microcracks or craze lines to appear within the root dentin. The severity attributed to these defects may depend on the flute form, pitch, taper, tip design, cross-sectional configuration, and taper of NiTi instruments [13,14].

In the research conducted by Kim et al., the prevalence of dentinal defects and canal irregularities was associated with a higher apical stress and strain gradient during instrumentation. Considering that root canal obturation and final restoration may initiate fractures or give rise to them to extend from such defects, these in turn contribute to an increased susceptibility to vertical root fracture [15,16].

The instrument's interaction with the dentin walls while the canal is being prepared shapes the canal. Numerous transient stress concentrations are produced by these interactions in the dentin. Such stress accumulations might lead to dentinal defects where vertical root fracture (VRF) may commence. Dentinal defects will likely rise as a result of higher forces placed on the root during instrumentation, which will also raise the likelihood of VRF [17].

TRN files caused fewer dentinal cracks than Neoendo Flex files, followed by Neoendo Neohybrid files, according to the results of the current investigation. TRN has an off-centered parallelogram cross-section design. It has been argued that TRN instruments maintain the remaining dentine and tooth intactness due to the instrument design, regressive tapers, and thinned design, along with the heat treatment of the NiTi alloy [13].

Neoendo Flex files have excellent cyclic fatigue resistance. The triangular cross-section with sharp cutting edges increases cutting efficiency. Avoiding accidental apical transportation becomes easier with the safety tip (non-cutting). Therefore, it causes fewer microcracks [18].

Neoendo Neohybrid offers a unique off-centered rectangular cross-section. There was minimal engagement between the file and canal dentin, effectively reducing taper-lock and screw-in forces as the file moves inside the canal. Less cross-sectional area creates higher flexibility for shaping severely curved canals and enhances loading and augering of debris. They have CTA wire-controlled thermal activation wire, which improves flexibility while retaining cutting efficiency. Therefore, it causes comparatively more cracks than other rotary files in this study [19].

Many endodontic complications can potentially occur when preparing the apical third of a canal, and as such, many techniques have been developed and clinically shown to manage these iatrogenic events. The apical third of the root canal system is typically a more challenging region to prepare because of its histological composition and anatomy. Anatomically, this region of the canal is tighter and more curved, and divisions are commonly present. This would be the reason for generating more stresses in the apical third of the root canal, which results in more dentinal microcrack formation [20].

NiTi rotary instruments deliver rotational stresses to the root canals, causing craze lines or microcracks to appear in the root dentin. In the present investigation, the emergence of such a defect may be influenced by the flute form, cross-sectional geometry, taper type (continuous or progressive), tip design, and pitch (constant or variable).

Limitations of the study

The possible limitations of this in vitro investigation were the application of elastomeric material to simulate the periodontal ligament. The elastomeric material may collapse and permit direct tooth-to-acrylic contact. Moreover, the clinical situation is more complex because the presence of periodontal ligament influences the distribution of stresses.

## Conclusions

It is possible to draw the conclusion that all of the investigated systems created dentinal cracks with respect to the limitations of this in vitro investigation. Neoendo Neohybrid file group had the most root dentinal defects, and Neoendo Flex file group came in second. The TruNatomy file group and control group showed significantly fewer root dentinal defects. Thus, a decision must be taken by the clinician considering the root dentin thickness and root canal anatomy to correspondingly use the suitable file system. To get more in-depth information, further studies need to be conducted.
